# Effective Chemical Inactivation of Ebola Virus

**DOI:** 10.3201/eid2207.160233

**Published:** 2016-07

**Authors:** Elaine Haddock, Friederike Feldmann, Heinz Feldmann

**Affiliations:** National Institute of Allergy and Infectious Diseases, National Institutes of Health, Hamilton, MT, USA

**Keywords:** Ebola virus, chemical inactivation, tissue culture, mouse model, viruses

## Abstract

Reliable inactivation of specimens before removal from high-level biocontainment is crucial for safe operation. To evaluate efficacy of methods of chemical inactivation, we compared in vitro and in vivo approaches using Ebola virus as a surrogate pathogen. Consequently, we have established parameters and protocols leading to reliable and effective inactivation.

The safe operation of high-level biocontainment laboratories throughout the world is of highest importance. These laboratories are under stringent national oversight and must adhere to international guidelines. Laboratories in the United States that handle select agents are further regulated by the US Centers for Disease Control and Prevention’s Division of Select Agents and Toxins and the US Department of Agriculture’s Animal and Plant Health Inspection Service.

Proper and reliable inactivation of specimens destined for removal from high-level biocontainment is a critical aspect for laboratory certification and operation. Standard operating procedures (SOPs) are approved by institutional biosafety committees in most cases and additionally by state and/or national regulatory authorities in other cases. In the past, specimens were commonly inactivated on the basis of operational experiences rather than well-documented protocols (*1*–*3*).

To evaluate the efficacy of chemical inactivation procedures for specimen removal, we used the US prime select agent and Tier-1 pathogen (*4*) *Zaire ebolavirus* (EBOV) as a surrogate model for enveloped high-level containment viruses with single-strand, negative-sense RNA genomes, such as arenaviruses, bunyaviruses, filoviruses, orthomyxoviruses, and paramyxoviruses. These viruses share certain biologic, biochemical, and structural features, making them sensitive to the same chemical inactivation methods. Furthermore, EBOV is currently a prominent example as the causative agent of an unprecedented epidemic in West Africa (*5*,*6*).

## The Study

Standard biologic specimens containing infectious EBOV commonly generated in high-level biocontainment operations were inactivated by several methods of chemical treatment (Figure; Table; Technical Appendix). For in vitro testing, we used wild-type EBOV expressing enhanced green fluorescent protein (EBOV-eGFP) (*7*), which allows for cytopathic effect (CPE) and fluorescence as simple readout parameters. For in vivo testing, we used mouse-adapted EBOV (MA-EBOV) (*8*) infection of BALB/c mice. Virus stocks were grown in Vero E6 cells and titrated by using a 50% tissue culture infectious dose (TCID_50_) assay (*9*). Infected cells were produced by infecting Vero E6 cells at a multiplicity of infection of 0.01. Cells were harvested at CPE of ≈75%, pelleted, and resuspended in 6 mL Dulbecco’s phosphate-buffered saline (DPBS); 1 mL aliquots were stored at −80°C. Samples were chemically treated according to the specific testing parameters and dialyzed or run over detergent-removal columns to remove inactivating reagents. In brief, samples were dialyzed by using a 10-kDa molecular weight cutoff (Spectrum Laboratories, Lawrenceville, GA, USA, or Fisher Scientific, Pittsburgh, PA, USA) and using DPBS over a stir plate at 4°C (>500-fold exchange volumes, 5 changes over 32–48 h); detergent was removed by using DetergentOUT GBS10–5000 columns (G-Biosciences, St. Louis, MO, USA). 

**Figure:**
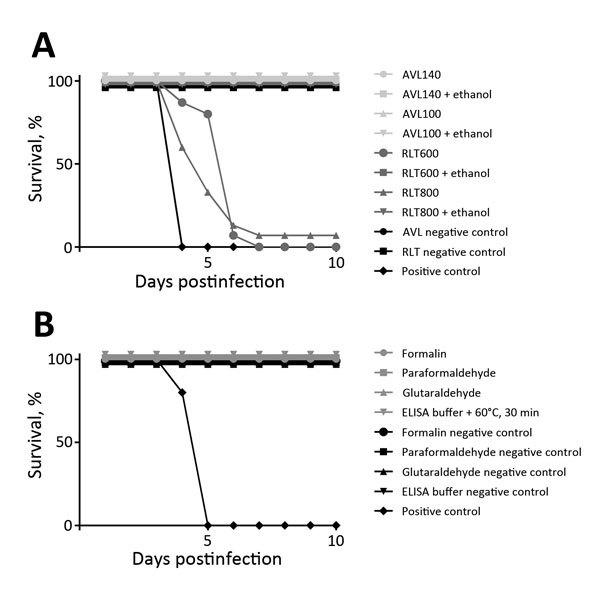
Ebola virus inactivation results as tested in BALB/c mouse model. A) Survival in animal groups tested with samples inactivated by guanidinium isothiocyanate buffers. AVL140, 140 µL Buffer AVL (QIAGEN, Valencia, CA, USA) + 560 µL sample; AVL100, 100 µL Buffer AVL + 600 µL sample; RLT600, 600 µL Buffer RLT (QIAGEN) treatment of cells; RLT800, 800 µL Buffer RLT treatment of cells; + ethanol, after a Buffer AVL or Buffer RLT inactivation contact time of 10 min, addition of 100% or 70% ethanol, respectively, for an additional 20 min of contact time. B) Survival in animal groups tested with samples inactivated by fixative or detergent buffers. For all test groups, n = 15; for all control groups, n = 5.

**Table:** Summary of methods and results for chemical inactivation of Ebola virus*

Inactivation method	Reagent volume	Sample type†	Inactivated sample (final viral load)	Contact time	Temp.	Reagent removal process	Result (in vitro)	Result (in vivo)
Buffer AVL	560 µL	Liquid virus stock	140 µL (1.4 × 10^6^ TCID_50_)	10 min	20°C	Dialysis	Pos (1/9)	Neg (0/15)
	600 µL	Liquid virus stock	100 µL (10^6^ TCID_50_)	10 min	20°C	Dialysis	Pos (1/9)	Neg (0/15)
	560 µL	Liquid virus stock	140 µL (1.4 × 10^6^ TCID_50_)	Overnight	4°C	Dialysis	Neg (0/9)	ND
	560 µL	Liquid virus stock	140 µL (1.4 × 10^6^ TCID_50_)	7 d	–80°C	Dialysis	Neg (0/9)	ND
Buffer AVL + ethanol‡	560 µL	Liquid virus stock	140 µL (1.4 × 10^6^ TCID_50_)	10 min + 20 min	20°C	Dialysis	Neg (0/9)	Neg (0/15)
	600 µL	Liquid virus stock	100 µL (10^6^ TCID_50_)	10 min + 20 min	20°C	Dialysis	Neg (0/9)	Neg (0/15)
Buffer RLT	600 µL	Cell pellet	5 × 10^6^ infected cells (≈5 × 10^6^ TCID_50_)	10 min	20°C	Dialysis	Pos (4/9)	Pos (15/15)
	800 µL	Cell pellet	5 × 10^6^ infected cells (≈5 × 10^6^ TCID_50_)	10 min	20°C	Dialysis	Pos (6/9)	Pos (14/15)
Buffer RLT + ethanol	600 µL	Cell pellet	5 × 10^6^ infected cells (≈5 × 10^6^ TCID_50_)	10 min + 20 min	20°C	Dialysis	Neg (0/9)	Neg (0/15)
	800 µL	Cell pellet	5 × 10^6^ infected cells (≈5 × 10^6^ TCID_50_)	10 min + 20 min	20°C	Dialysis	Neg (0/9)	Neg (0/15)
	600 µL	Tissue	30 mg (≈3 × 10^5^ TCID_50_)	10 min + 20 min	20°C	Dialysis	Neg (0/9)	ND
TRIzol§	750 µL, 75% final	Cell pellet in 250 µL	5 × 10^6^ infected cells (≈5 × 10^6^ TCID_50_)	10 min	20°C	Dialysis	Neg (0/9)	ND
	750 µL, 75% final	Blood	250 µL (≈2.5 × 10^5^ TCID_50_)	10 min	20°C	Dialysis	Neg (0/9)	ND
	1 mL	Tissue	50 mg (≈5 × 10^5^ TCID_50_)	10 min	20°C	Dialysis	Neg (0/9)	ND
Formalin	750 µL, 7.5% final	Cells, 250 µL	2.5 × 10^6^ infected cells (≈2.5 × 10^6^ TCID_50_)	Overnight	4°C	Dialysis	Neg (0/9)	Neg (0/15)
	10 mL, 10% final	Tissue	150 mg (≈1.5x^6^ TCID_50_)	7 d or 30 d¶	4°C	Dialysis	Neg (0/9)	ND
Glutaraldehyde	1.3 mL, 2% final	Cells, 330 µL	3.3 × 10^6^ infected cells (≈3.3 × 10^6^ TCID_50_)	Overnight	4°C	Dialysis	Neg (0/9)	Neg (0/15)
	10 mL, 2% final	Tissue	150 mg (≈1.5 × 10^6^ TCID_50_)	7 d	4°C	Dialysis	Neg (0/9)	ND
Paraformaldehyde	1.3 mL, 2% final	Cells, 330 µL	3.3 × 10^6^ infected cells (≈3.3 × 10^6^ TCID_50_)	Overnight	4°C	Dialysis	Neg (0/9)	Neg (0/15)
	10 mL, 2% final	Tissue	150 mg (≈1.5x^6^ TCID_50_)	7 d	4°C	Dialysis	Neg (0/9)	ND
Heat#	NA	Cells	1 mL, 1:10 dilution (≈10^6^ TCID_50_)	5 min	100°C	NA	Pos (3/3)	ND
	NA	Cells	1 mL, 1:10 dilution (≈10^6^ TCID_50_)	10 min	100°C	NA	Neg (0/9)	ND
	NA	Cells	1 mL, 1:10 dilution (≈10^6^ TCID_50_)	5 min	120°C	NA	Neg (0/9)	ND
	NA	Cells	1 mL, 1:10 dilution (≈10^6^ TCID_50_)	10 min	120°C	NA	Neg (0/9)	ND
	NA	Liquid virus stock	1 mL, 1:10 dilution (10^6^ TCID_50_)	15 min	65°C or 70°C	NA	Pos (6/6)	ND
	NA	Liquid virus stock	1 mL, 1:10 dilution (10^6^ TCID_50_)	30 min	60°C or 65°C	NA	Pos (6/6)	ND
ELISA buffer	960 µL	Liquid virus stock	40 µL (4 × 10^5^ TCID_50_)	10 min	20°C	Detergent column	Pos (2/9)	ND
Heat + ELISA buffer	960 µL	Liquid virus stock	40 µL (4 × 10^5^ TCID_50_)	30 min	60°C	Detergent column	Neg (0/9)	Neg (0/15)
	960 µL	Liquid virus stock	40 µL (4 × 10^5^ TCID_50_)	15 min	65°C	Detergent column	Neg (0/9)	ND
	960 µL	Liquid virus stock	40 µL (4 × 10^5^ TCID_50_)	30 min	65°C	Detergent column	Neg (0/9)	ND
	960 µL	Liquid virus stock	40 µL (4 × 10^5^ TCID_50_)	15 min	70°C	Detergent column	Neg (0/9)	ND
1% SDS, 5% 2-ME**	250 µL, 4×	Cells, 250 µL	2.5 × 10^6^ infected cells (≈2.5 × 10^6^ TCID_50_)	10 min	20°C	Detergent column	Neg (0/9)	ND
	250 µL, 4×	Tissue	150 mg (≈1.5 × 10^6^ TCID_50_)	10 min	20°C	Detergent column	Neg (0/9)	ND
1% SDS	250 µL, 4×	Cells, 250 µL	2.5 × 10^6^ infected cells (≈2.5 × 10^6^ TCID_50_)	10 min	20°C	Detergent column	Neg (0/9)	ND

*DPBS, Dulbecco’s phosphate-buffered saline; NA, non-applicable; ND, not done; SDS, sodium dodecyl sulfate; TCID_50_, tissue culture infectious dose 50. †Initial sample virus titers were as follows: liquid virus stock (1 × 10^7^ TCID_50_/mL), infected cells (≈1 × 10^7^ TCID_50_/mL, 1 × 10^7^ cells/mL), blood (≈10^6^ TCID_50_/mL), tissue (≈10^4^ TCID_50_/mg). Infected cells (5 × 10^6^ cells) were pelleted by centrifugation and lysed in test reagent (Buffer RLT; QIAGEN, Valencia, CA, USA), pelleted and resuspended in 250 μL DPBS before addition of test reagent (TRIzol; Life Technologies, Grand Island, NY, USA), or used directly in solution at the concentration and volume described in the table (fixative, heat, and SDS testing). Ebola virus–infected blood and liver tissue were collected from BALB/c mice infected with mouse-adapted Ebola virus at the height of infection, or from mock infected mice at a corresponding day. ‡Addition of ethanol: after contact time with Buffer AVL (QIAGEN, Valencia, CA, USA) or Buffer RLT, samples were transferred to a clean tube with 560 μL 100% ethanol (for AVL inactivation) or 600 μL 70% ethanol (for RLT inactivation) and allowed an additional 20 minutes contact time at 20°C. §Although Spectra/Por Float-A-Lyzer G2 dialysis tubes (Spectrum Laboratories, Lawrenceville, GA, USA) were used for all other reagent removal by dialysis, Slide-A-Lyzer cassettes (Fisher Scientific, Pittsburgh, PA, USA) were used with TRIzol because the cassettes are more resistant to direct degradation by the reagent. ¶Tissue samples <1 cm^3^ were treated with a 7-day contact time, whereas samples >1 cm^3^ were treated with a 30-day contact time. #Samples were boiled in an AccuBlock Digital Dry Bath (Sigma-Aldrich, St. Louis, MO, USA). All other heat treatments were carried out in a nonshaking water bath. **4× SDS loading buffer contains 200 mmol/L Tris (pH 6.8), 4% SDS, 35% glycerol, 0.05% bromophenol blue and 20% 2-ME (added at the time of use). A combination of 250 μL 4× buffer, sample, and DPBS for a combined volume of 1 mL was tested; an appropriate volume of DPBS was substituted for 2-ME as necessary for testing of SDS as the single inactivating reagent.

Negative control samples included DPBS and noninfected Vero E6 cells and tissue homogenates (mouse); positive control samples included untreated virus stocks and infected Vero E6 cells and mouse tissues. For in vitro testing, all samples were increased in volume to 3 mL and equally divided to infect Vero E6 cells (80% confluency) in triplicates. Cells were incubated at 37°C for 14 days and monitored regularly for CPE or fluorescence. For in vivo testing, samples were increased in volume to 1 mL and equally divided to infect 5 mice intraperitoneally. BALB/c mice (female, 6–8 weeks old; Charles River Laboratories, Wilmington, MA, USA) were housed in microisolator cages and were monitored daily for 28 days. Because in vitro and in vivo safety testing correlated well, we discontinued mouse infections for ethical reasons.

Nucleic acid extraction is often carried out with commercial guanidinium isothiocyanate buffers. We used Buffer AVL and Buffer RLT (QIAGEN, Valencia, CA, USA) and TRIzol (Life Technologies, Grand Island, NY, USA) according to manufacturers’ recommendations. AVL was mixed with stock virus at different ratios, and infected cells were resuspended in RLT (Table). Samples were either immediately dialyzed or treated with ethanol (AVL, 100% ethanol, 560 μL; RLT, 70% ethanol, 600 μL). Infected liver tissue was homogenized in RLT with a stainless steel bead (10 min at 30 Hz). A soluble aliquot (≈30 mg) was transferred to a new tube, and fresh RLT was added, followed by 70% ethanol (600 μL). After dialysis, samples were used to infect Vero E6 cells and mice. Similar to a results in a previous study (*10*), AVL and RLT treatment alone for 10 minutes at either ratio did not fully inactivate EBOV; however, the addition of ethanol (the next step of the manufacturer’s protocol) rendered all samples completely noninfectious. AVL alone resulted in complete inactivation with longer contact times (i.e., refrigerated overnight or frozen for 7 days) (Table; Figure).

Infected cells were resuspended and treated with TRIzol (1:4 vol/vol). Infected liver samples were homogenized in 1 mL TRIzol as described in the previous paragraph. After centrifugation, an aliquot of tissue homogenate (≈50 mg) was transferred to a new tube, and fresh TRIzol was added. Additionally, blood from infected animals was mixed (1:4 vol/vol) with TRIzol. After dialysis, Vero E6 cells were inoculated and monitored for CPE or fluorescence. In all cases, virus growth was not detected (Table), indicating complete inactivation.

Formalin, paraformaldehyde, and glutaraldehyde can be used to fix cells or tissues for histologic or microscopic studies. Infected cells were diluted 1:4 in 10% neutral-buffered formalin (7.5% fixative) or 1:5 in either 2.5% glutaraldehyde or 2.5% paraformaldehyde (2% fixative). Samples were dialyzed and used to infect Vero E6 cells or mice. Monitoring of cell culture and animals resulted in the absence of CPE or fluorescence and clinical signs, respectively, indicating complete inactivation of EBOV (Table; Figure).

Infected liver segments were incubated in 10% neutral-buffered formalin, 2% glutaraldehyde, or 2% paraformaldehyde (10 mL) for a period of 7 days (<1-cm^3^ piece) or 30 days (>1-cm^3^ piece) at 4°C. Subsequently, a small section of tissue (≈150 mg) was dissected, homogenized in DPBS with a stainless steel bead (10 min at 30 Hz), and then dialyzed. After dialysis, samples were used to infect Vero E6 cells. All samples were completely inactivated (Table).

Samples for protein assays are often inactivated by a combination of detergent and heat. We tested the parameters of 60°C for 30 min, 65°C for 15 or 30 min, and 70°C for 15 min in conjunction with a buffer containing 0.5% Triton X-100 and 0.5% Tween-20 (both from Sigma-Aldrich, St. Louis, MO, USA); this mixture is commonly used for ELISA. Stock virus was diluted 1:25 in this buffer and heated for the appropriate times before samples were clarified of detergent and used to infect Vero E6 cells or mice. All samples were completely inactivated as indicated by lack of CPE or fluorescence in cells and clinical signs in mice (Table; Figure).

Boiling (at 100°C for 10 min or 120°C for 5 min) might be sufficient to inactivate EBOV (Table) (*11*) but is often used in conjunction with sodium dodecyl sulfate (SDS)–containing buffers for protein analysis. Aliquots of infected cells were diluted in DPBS and 4× loading buffer (1% SDS final). Infected liver tissue (≈150 mg) were placed in DPBS and 4× loading buffer (1% SDS final). The samples were then homogenized with a stainless steel bead (10 min at 30 Hz). After detergent removal, samples were used to infect Vero E6 cells; all treated cells and tissue homogenates were negative for infectious EBOV (Table).

## Conclusions

Our study establishes inactivation procedures for EBOV that can be safely applied to distinct specimen types and research purposes and might also apply to other enveloped, single-strand, negative-sense RNA viruses. Our findings should help to improve and approve SOPs for inactivation without the need for safety testing each individual sample, an unfeasible and unwarranted task in current diagnostic and research operations in high-level biocontainment settings. However, any changes to inactivation SOPs make further safety testing essential. Safety testing for inactivation, at least for EBOV, can rely on cell culture only because this seems to be as sensitive as in vivo testing.

**Technical Appendix.** Materials and methods used to establish effective chemical inactivation of Ebola virus.
