# Supplementary material for: Effective Chemical Inactivation of Ebola Virus
Source: Emerg Infect Dis. 2016 Jul;22(7):1292–4. doi: 10.3201/eid2207.160233 (PMC4918181; doi:10.3201/eid2207.160233)
Supplement: Supplementary file 1 — Technical Appendix. Materials and methods used to establish effective chemical inactivation of Ebola virus. [file 16-0233-Techapp-s1.pdf]

Article DOI: http://dx.doi.org/10.3201/eid2207.160233

Effective Chemical Inactivation of Ebola Virus

Technical Appendix

Materials and Methods

Ethics and Safety Statement

All work involving infectious material was performed in the Biosafety Level 4 (BSL4) laboratory at Rocky Mountain Laboratories (RML), National Institute of Allergy and Infectious Diseases (NIAID), National Institutes of Health (NIH). Animal experiments were approved by the Institutional Animal Care and Use Committee (IACUC) of RML and performed following the guidelines of the Association for Assessment and Accreditation of Laboratory Animal Care, International (AAALAC), by certified staff in an AAALAC-approved facility.

Viruses and Infected Cells

Testing involved 10^7 FFU/ mL stock virus of wild-type EBOV expressing enhanced green fluorescent protein (EBOV-eGFP) or 10^7 FFU/ mL stock virus of mouse-adapted EBOV (MA-EBOV). Virus stocks were grown in VERO E6 cells and titrated using a focus forming unit (FFU) assay. Infected cells were produced by infecting VERO E6 cells at a multiplicity of infection of 0.01. Cells were harvested at CPE of ≈75%, pelleted, resuspended in 6 mL Dulbecco’s phosphate-buffered saline (DPBS) and 1 mL aliquots were stored at $-80^{o}C$ until use (1×10^7 cells/ mL). Negative control cells were made of similarly treated, uninfected cell monolayers. For those tests involving blood or tissues, BALB/c mice were infected with MA-EBOV and blood and liver were collected at the height of disease. Blood and liver from uninfected control animals were used as negative control samples.

Inactivation Reagents

Buffers AVL and RLT (Qiagen), TRIzol (Life Technologies), 10% neutral-buffered formalin (Leica Biosystems), paraformaldehyde (Electron Microscopy Science) and glutaraldehyde (Sigma Aldrich) were tested as supplied for RNA and fixation samples. A sodium

dodecyl sulfate (SDS) 4× loading buffer (200 mmol/L Tris, pH 6.8; 4% SDS; 35% glycerol; 0.05% bromophenol blue; 20% 2-mercaptoe thanol (added at time of use)) and an enzyme-linked immunosorbent assay (ELISA) buffer (DPBS with 5% skim milk; 0.5% Triton X-100; 0.5% Tween-20) were produced in-house for protein samples.

Dialysis

For those tests requiring dialysis, samples were dialyzed with a 10kDa molecular weight cutoff. DPBS was used as a dialysis buffer at >500-fold exchange volumes over five changes and 32-48 hours, at 4°C over a stir plate, before samples were collected and used to infect for testing. All samples were dialyzed using Spectra/Por Float-A-Lyzer G2 tubing (Spectrum Laboratories) with the exception of those involving TRIzol, which was dialyzed using Slide-A-Lyzer cassettes (Fisher Scientific).

Detergent Removal

DetergentOUT GBS10-5000 columns (G-Biosciences) were utilized to remove detergent from samples, per manufacturer's recommended protocol. Columns were equilibrated twice with DPBS before the detergent-containing sample was incubated on the column for two minutes, spun through the column, collected and used to infect for testing.

Validation Protocol, Cell Culture Model

Virus-infected samples (in triplicate unless otherwise noted) were treated according to the specific testing parameters and dialyzed or run over detergent-removal columns to remove inactivating reagents. Each of the treated samples was then increased in volume to 3 mL as necessary and equally divided to infect triplicate 25 cm^2 flasks of fresh VERO E6 cells at 80% confluency. Following an infection time of 60 minutes at 37°C, inoculum was removed and 6 mL/flask DMEM with 2% FBS was added. Unless otherwise noted, cells were not washed before addition of fresh medium. Cells were incubated at 37°C for an additional 14 days and monitored regularly for CPE (MA-EBOV) or CPE and fluorescence (EBOV-eGFP). Positive and negative samples were included in every validation, subjected to the same mechanical treatments (e.g. dialysis, spin columns) as the test samples, and tested on 3 flasks of fresh cells each. Negative control samples were DPBS or uninfected cells /tissue homogenates from animals which were not infected; positive control samples were virus stock/infected cells/infected tissue homogenates

that were not treated. A DPBS mock infection of 3 flasks was included in each experiment to control for residual inactivating reagent.

Validation Protocol, Animal Model

Six- to eight-week old female BALB/c mice (Charles River Laboratories) were housed in microisolator cages and allowed to acclimatize prior to use in experiments. Three virus-infected samples were treated according to the specific testing parameters and dialyzed or run over detergent-removal columns to remove inactivating reagents. Each of the treated samples was then increased in volume to 1 mL as necessary and equally divided to infect 5 mice intraperitoneally. Mice were assessed daily according to approved protocol for weight loss and/or other clinical signs of illness for 28 days post infection. Positive and negative samples were included in every validation, subjected to the same mechanical treatments (e.g. dialysis, spin columns) as the test samples, and tested in groups of 5 animals each. Negative control samples were DPBS or uninfected cells; positive control samples were virus stock/infected cells that were not treated. A DPBS mock infection of 5 animals was included in each experiment to control for residual inactivating reagent.

Protocols

Buffer AVL Testing

140 µL Liquid virus stock (1.4×10^6 $TCID_{50}$)

\+ 560 µL Buffer AVL

10 minute contact time in 2 mL tube at 20°C. Dialyzed in Spectra/Por Float-A-Lyzer tubes.

Or

140 µL Liquid virus stock (1.4×10^6 $TCID_{50}$)

\+ 560 µL Buffer AVL

10 minute contact time in 2 mL tube at 20°C. Then transferred to 2 mL tube containing 560 µL 100% ethanol for 20 minutes at 20°C. Dialyzed in Spectra/Por Float-A-Lyzer tubes.

Or

100 µL Liquid virus stock (1.0 × 10^6 $TCID_{50}$)

\+ 600 µL Buffer AVL

10 minute contact time in 2 mL tube at 20°C. Dialyzed in Spectra/Por Float-A-Lyzer tubes.

Or

100 µL Liquid virus stock (1.0 × 10^6 $TCID_{50}$)

\+ 600 µL Buffer AVL

10 minute contact time in 2 mL tube at 20°C. Then transferred to 2 mL tube containing 560 µL 100% ethanol for 20 minutes at 20°C. Dialyzed in Spectra/Por Float-A-Lyzer tubes.

Or

140 µL Liquid virus stock (1.4 × 10^6 $TCID_{50}$)

\+ 560 µL Buffer AVL

Overnight contact time in 2 mL tube at 4°C. Dialyzed in Spectra/Por Float-A-Lyzer tubes.

Or

140 µL Liquid virus stock (1.0 × 10^6 $TCID_{50}$)

\+ 560 µL Buffer AVL

7 day contact time in 2 mL tube at –80°C. Dialyzed in Spectra/Por Float-A-Lyzer tubes.

All samples were removed from dialysis tubing, raised to 3 mL final volume with DPBS, and split evenly to infect three flasks of VERO E6 cells. Infection contact time was 1 hour; inoculum was removed and replaced with medium (DMEM). Cells were not washed. For those methods tested in mice, samples were raised to an equal volume (≈1.4 mL) with DPBS and split equally to infect 5 mice.

Buffer RLT Testing

Infected cells pelleted and supernatant removed (5 × 10^6 infected cells, ≈5 × 10^6 $TCID_{50}$)

\+ 600 µL Buffer RLT

10 minute contact time in 2 mL tube at 20°C. Dialyzed in Spectra/Por Float-A-Lyzer tubes.

Or

Infected cells pelleted and supernatant removed (5×10^6 infected cells, ≈5×10^6 $TCID_{50}$)

<u>+ 600 µL Buffer RLT</u>

10 minute contact time in 2 mL tube at 20°C. Then transferred to 2 mL tube containing 600 µL 70% ethanol for 20 minutes at 20°C. Dialyzed in Spectra/Por Float-A-Lyzer tubes.

Or

Infected cells pelleted and supernatant removed (5×10^6 infected cells, ≈5×10^6 $TCID_{50}$)

<u>+ 800 µL Buffer RLT</u>

10 minute contact time in 2 mL tube at 20°C. Dialyzed in Spectra/Por Float-A-Lyzer tubes.

Or

Infected cells pelleted and supernatant removed (5×10^6 infected cells, ≈ 5×10^6 $TCID_{50}$)

<u>+ 800 µL Buffer RLT</u>

10 minute contact time in 2 mL tube at 20°C. Then transferred to 2 mL tube containing 600 µL 70% ethanol for 20 minutes at 20°C. Dialyzed in Spectra/Por Float-A-Lyzer tubes.

Or

30 mg infected liver (≈3×10^5 $TCID_{50}$)

<u>+ 600 µL Buffer RLT</u>

A larger piece (≈100 mg) was homogenized in 600 µL Buffer RLT at 30Hz with a stainless steel bead for 10 minutes in a2 mL tube at 20°C. The equivalent volume of 30 mg (180 µL) was transferred to a clean 2 mL tube and a volume of Buffer RLT (320 µL) added to bring volume back to 600 µL total. This was followed by a 10 minute contact time at 20°C, after which the entire sample was transferred to 2 mL tube containing 600 µL 70% ethanol for 20 minutes at 20°C. Dialyzed in Spectra/Por Float-A-Lyzer tubes.

All samples were removed from dialysis tubing, raised to 3 mL final volume with DPBS, and split evenly to infect three flasks of VERO E6 cells. Infection contact time was 1 hour;

inoculum was removed and replaced with medium (DMEM). Cells were not washed. For those methods tested in mice, samples were raised to an equal volume (≈1.4 mL) with DPBS and split equally to infect 5 mice.

TRIzol Testing

Infected cells pelleted and resuspended in 250 µL DPBS (≈5 × 10^6 infected cells, 5 × 10^6 $TCID_{50}$)

+ 750 µL TRIzol

10 minute contact time in 2 mL tube at 20°C. Dialyzed in Slide-A-Lyzer tubes.

Or

250 µL infected blood (≈2.5 × 10^5 $TCID_{50}$)

+ 750 µL TRIzol

10 minute contact time in 2 mL tube at 20°C. Dialyzed in Slide-A-Lyzer tubes.

Or

50 mg infected liver (5 × 10^5 $TCID_{50}$)

+ 1 mL TRIzol

A larger piece (≈100 mg) was homogenized in 1 mL TRIzol at 30Hz with a stainless steel bead for 10 minutes in a 2 mL tube at 20°C. The equivalent volume of 50 mg (0.5 mL) was transferred to a clean 2 mL tube and a volume of TRIzol (0.5 mL) added to bring volume back to 1 mL total. This was followed by a 10 minute contact time in 2 mL tube at 20°C. Dialyzed in Slide-A-Lyzer tubes.

All samples were removed from dialysis tubing, raised to 3 mL final volume with DPBS, and split evenly to infect three flasks of VERO E6 cells. Infection contact time was 1 hour; inoculum was removed, cells were washed to remove any traces of TRIzol and medium (DMEM) was added. TRIzol samples were not tested in mice.

Formalin Testing

250 µL infected cells (2.5 × 10^6 infected cells, ≈2.5 × 10^6 $TCID_{50}$)

+ 750 μL 10% formalin (7.5% final concentration)

Overnight contact time in 2 mL tube at 4°C. Dialyzed in Spectra/Por Float-A-Lyzer tubes.

Or

One-half infected liver lobe

+ 10 mL 10% formalin

7 day contact time in 15 mL tube at 4°C

This larger piece was dissected following contact time for a smaller internal piece (150 mg, ≈1.5 × 10^6 $TCID_{50}$) which was homogenized in 1 mL DPBS at 30Hz with a stainless steel bead for 10 minutes in a 2 mL tube at 20°C. Dialyzed in Spectra/Por Float-A-Lyzer tubes.

Or

One infected liver

+ 10 mL 10% formalin

30 day contact time in 15 mL tube at 4°C

This larger piece was dissected following contact time for a smaller internal piece (150 mg, ≈1.5 × 10^6 $TCID_{50}$) which was homogenized in 1 mL DPBS at 30Hz with a stainless steel bead for 10 minutes in a 2 mL tube at 20°C. Dialyzed in Spectra/Por Float-A-Lyzer tubes.

All samples were removed from dialysis tubing, raised to 3 mL final volume with DPBS, and split evenly to infect three flasks of VERO E6 cells. Infection contact time was 1 hour; inoculum was removed and replaced with medium (DMEM). Cells were not washed. For those methods tested in mice, samples were split equally to infect 5 mice.

Glutaraldehyde and Paraformaldehyde Testing

330 μL infected cells (3.3 × 10^6 infected cells, ≈3.3 × 10^6 $TCID_{50}$)

+ 1.3 mL 2.5% Glutaraldehyde (2% final concentration)

overnight contact time in 2 mL tube at 4°C. Dialyzed in Spectra/Por Float-A-Lyzer tubes.

Or

330 μL infected cells (3.3 × 10^6 infected cells, ≈3.3 × 10^6 $TCID_{50}$)

+ 1.3 mL 2.5% Paraformaldehyde (2% final concentration)

overnight contact time in 2 mL tube at 4°C. Dialyzed in Spectra/Por Float-A-Lyzer tubes.

Or

One-half infected liver lobe

+ 10 mL 2% Glutaraldehyde

7 day contact time in 15 mL tube at 4°C

This larger piece was dissected following contact time for a smaller internal piece (150 mg, ≈1.5 × 10^6 $TCID_{50}$) which was homogenized in 1 mL DPBS at 30Hz with a stainless steel bead for 10 minutes in a 2 mL tube at 20°C. Dialyzed in Spectra/Por Float-A-Lyzer tubes.

Or

One-half infected liver lobe

+ 10 mL 2% Paraformaldehyde

7 day contact time in 15 mL tube at 4°C

This larger piece was dissected following contact time for a smaller internal piece (150 mg, ≈1.5 × 10^6 $TCID_{50}$) which was homogenized in 1 mL DPBS at 30Hz with a stainless steel bead for 10 minutes in a 2 mL tube at 20°C. Dialyzed in Spectra/Por Float-A-Lyzer tubes.

All samples were removed from dialysis tubing, raised to 3 mL final volume with DPBS, and split evenly to infect three flasks of VERO E6 cells. Infection contact time was 1 hour; inoculum was removed and replaced with medium (DMEM). Cells were not washed. For those methods tested in mice, samples were split equally to infect 5 mice.

Heat Testing

1 mL 1:10 dilution of infected cells (1 × 10^6 infected cells, ≈1 × 10^6 $TCID_{50}$)

Heat 5 or 10 minutes in AccuBlock heater at 100°C or 120°C in 2 mL tubes

Or

1 mL 1:10 dilution of liquid stock virus (≈1 × 10^6 $TCID_{50}$)

Heat 15 or 30 minutes in non-shaking water bath at 60°C, 65°C or 70°C in 2 mL tubes

All samples were raised to 3 mL final volume with DPBS, and split evenly to infect three flasks of VERO E6 cells. Infection contact time was 1 hour; inoculum was removed and replaced with medium (DMEM). Cells were not washed. Heat samples were not tested in mice.

Detergent Testing

ELISA Buffer Testing

40 µL Liquid virus stock (4×10^5 $TCID_{50}$)

+ 960 µL ELISA Buffer (with 0.5% Triton X-100 and 0.5% Tween-20)

10 minute contact time in 2 mL tube at 20°C. Spun through pre-equilibrated (DPBS) DetergentOUT GBS10-5000 column.

Or

40 µL Liquid virus stock (4×10^5 $TCID_{50}$)

+ 960 µL ELISA Buffer (with 0.5% Triton X-100 and 0.5% Tween-20)

15 or 30 minute contact in non-shaking water bath at 60°C, 65°C or 70°C in 2 mL tubes. Spun through pre-equilibrated (DPBS) DetergentOUT GBS10-5000 column

SDS Buffer Testing

250 µL infected cells (2.5×10^6 cells, $\approx 2.5 \times 10^6$ $TCID_{50}$)

500 µL DPBS

+ 250 µL 4× SDS Loading Buffer (with 4% SDS and 20% 2-ME)

10 minute contact time in 2 mL tube at 20°C. Spun through pre-equilibrated (DPBS) DetergentOUT GBS10-5000 column.

Or

250 µL infected cells (2.5×10^6 cells, $\approx 2.5 \times 10^6$ $TCID_{50}$)

500 µL DPBS

+ 250 µL 4× SDS Loading Buffer (with 4% SDS, no 2-ME)

10 minute contact time in 2 mL tube at 20°C. Spun through pre-equilibrated (DPBS) DetergentOUT GBS10-5000 column.

Or

150 mg infected liver (≈1.5 × 10^6 $TCID_{50}$)

750 µL DPBS

\+ 250 µL 4× SDS Loading Buffer (with 4% SDS and 20% 2-ME)

Tissue was homogenized in 1 mL buffer with 1%SDS and 5% 2-ME at 30Hz with a stainless steel bead for 10 minutes in a 2 mL tube at 20°C. Spun through pre-equilibrated (DPBS) DetergentOUT GBS10-5000 column.

All samples were raised to 3 mL final volume with DPBS, and split evenly to infect three flasks of VERO E6 cells. Infection contact time was 1 hour; inoculum was removed, cells washed and medium (DMEM) was added. After incubation for 24 hours, half the medium was removed and replaced with and additional full volume to avoid a pH drop occasionally seen after use of detergent removal columns. For those methods tested in mice, samples were split equally to infect 5 mice.
